# A novel locomotion-based prepulse inhibition assay in zebrafish larvae

**DOI:** 10.17912/micropub.biology.000914

**Published:** 2024-01-24

**Authors:** Emily Read, Robert Hindges

**Affiliations:** 1 Centre for Developmental Neurobiology & MRC Centre for Neurodevelopmental Disorders, King's College London, London, England, United Kingdom

## Abstract

Sensory gating, measured using prepulse inhibition (PPI), is an endophenotype of neuropsychiatric disorders that can be assessed in larval zebrafish models. However, current PPI assays require high-speed cameras to capture rapid c-bend startle behaviours of the larvae. In this study, we designed and employed a PPI paradigm that uses locomotion as a read-out of zebrafish larval startle responses. PPI percentage was measured at a maximum of 87% and strongly reduced upon administration of the NMDA receptor antagonist, MK-801. This work provides the foundation for simpler and more accessible PPI assays using larval zebrafish to model key endophenotypes of neurodevelopmental disorders.

**Figure 1. Locomotion-based prepulse inhibition and modulation by MK-801:**
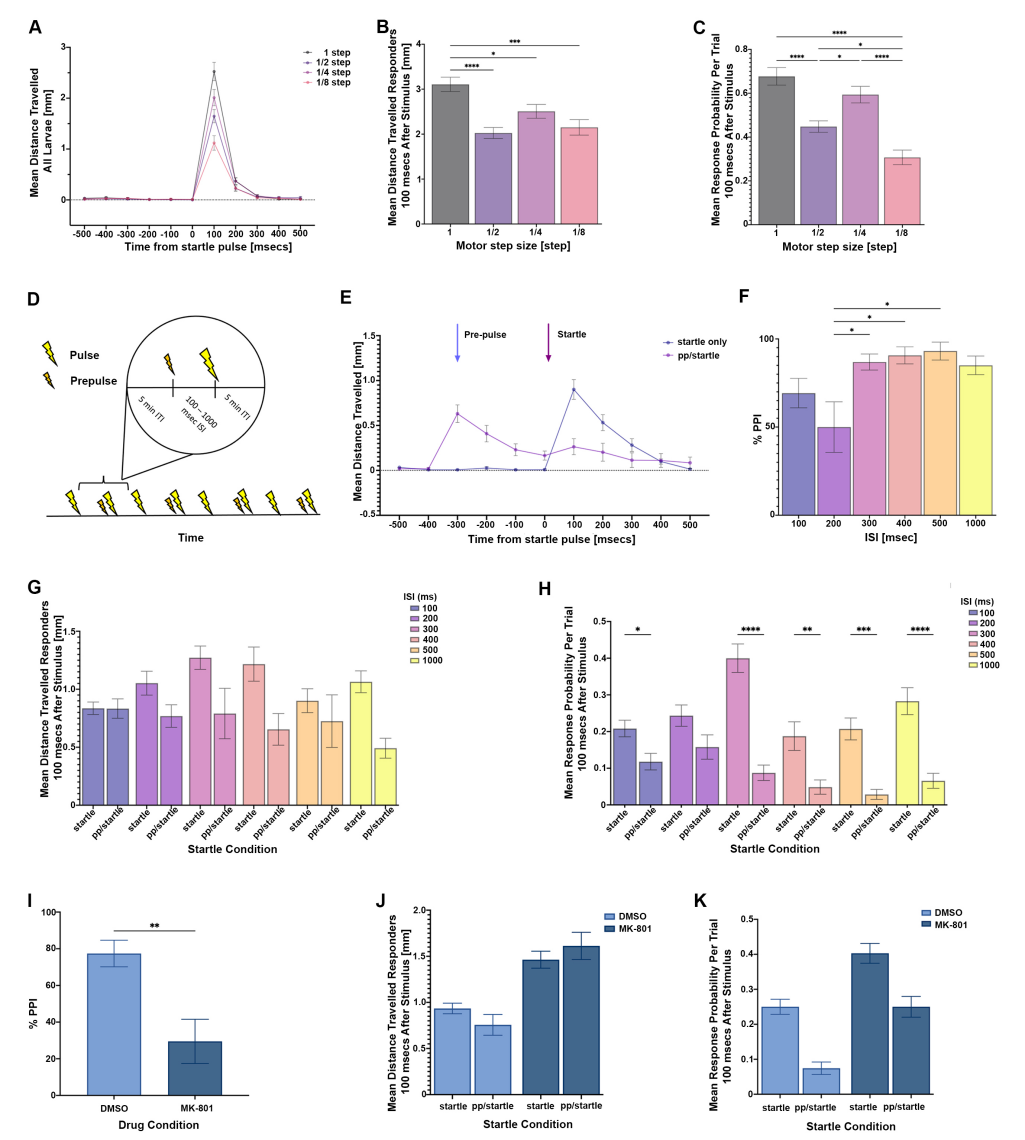
**A.** Locomotion trace showing the response of all larvae (6dpf) after within-subject presentation of eight 200 Hz 20 ms vibrations (two vibrations per motor step, randomised order). Data are presented as the average locomotion (mm) for each pulse intensity. **B.** Response magnitude of responder larvae. Data are presented as the average locomotion (mm) for each pulse intensity 100 ms after the startle stimulus. **C.** Response probability of all larvae. Data are presented as the average response probability per trial 100 ms after the startle stimulus. **D.** Diagram to show the finalised PPI assay procedure. **E.** 300 ms ISI raw locomotion data. Data are presented as the mean distance travelled (mm) by all larvae in startle pulse only (blue) and prepulse/startle (purple) trials. Arrows indicate time points where the prepulse (blue) and startle (purple) stimuli are delivered on relevant trials. **F.** Percentage PPI for responder larvae at ISIs of 100 – 1000 ms (for PPI calculation, see Methods). **G.** Response magnitude of responder larvae in startle alone and prepulse/startle trials across 100-1000 ms ISIs. Data are presented as average locomotion (mm) 100 ms after the startle stimulus. **H.** Response probability of all larvae across 100-1000 ms ISIs. Data are presented as average response probability per trial, 100 msec after the startle stimulus. **I.** Percentage PPI for responder larvae treated with MK-801 or a DMSO-control solution. **J.** Response magnitude of responder larvae treated with MK-801 or a DMSO-control solution in startle alone and prepulse/startle trials. Data are presented as average locomotion (mm) 100 ms after the startle stimulus. **K.** Response probability of all larvae treated with MK-801 or a DMSO-control solution in startle alone and prepulse/startle trials. Data are presented as average response probability per trial, 100 ms after the startle stimulus. For all *N* -numbers, see Table 1 in Methods section. * = *p* <0.05, ** = *p* <0.01, *** = *p* <0.001, **** = *p* <0.0001.

## Description

Impairments of sensory gating have been proposed as one of the most promising translational endophenotypes for various neuropsychological disorders, including schizophrenia. Sensory gating is defined as the ability of the autonomic nervous system to filter between relevant and irrelevant sensory information. Measurement of this endophenotype is via the prepulse inhibition (PPI) assay. Here, startle responses are reduced in trials when a lower intensity (prepulse) stimulus is presented 30-500 milliseconds (ms) prior to a higher intensity (startle) stimulus. Patients with schizophrenia and high-risk individuals show reliable PPI reductions (compared to healthy controls), maintaining a high startle response even if the startle stimulus is preceded by a prepulse stimulus (Li et al., 2021; San-Martin et al., 2020; Swerdlow et al., 2006; Takahashi & Kamio, 2018; Ziermans et al., 2012) . PPI is detected across different species, including common model organisms, and therefore the assay has become highly relevant to investigate mechanisms underlying the aetiology of disorders.

PPI responses can be measured in the zebrafish ( *Danio rerio* ). Using larval stages, the proportion of fish displaying a characteristic ‘c-bend’ startle response is reduced when the startle stimulus is preceded by a prepulse (Burgess & Granato, 2007) . The parameters required for PPI in zebrafish larvae are strikingly similar to that used in human PPI assays (Wolman et al., 2011) . Furthermore, pharmacological and genetic manipulations, that are used to model schizophrenia in zebrafish, also lead to reductions in the PPI response (Bergeron et al., 2015; Burgess & Granato, 2007) , consistent with the observations in patients.

However, the c-bend startle must be measured using high-speed cameras. There are two reasons for this. Firstly, the c-bend startle is defined by precise angular changes in the orientation of the larva’s tail with respect to its head (Bergeron et al., 2015) . Secondly, there are at least two c-bend startles shown by zebrafish larvae: the short-latency c-bend (SLC) and the long-latency c-bend (LLC) (Burgess & Granato, 2007) . The SLC is a probabilistic startle response, increasing in frequency with stimulus intensity (but not in amplitude), and occurring in just 10 ms or less. In contrast, the LLC startle is graded in amplitude and latency to stimulus intensities and occurs at a longer latency of 20-50 ms. Previous data show that only the SLC is susceptible to PPI (Burgess & Granato, 2007) . Taken together, a camera therefore needs to be able to image detailed larval features at a high temporal resolution to measure PPI. Due to the expense of high-speed camera equipment and the extensive data analysis to characterise SLC startles, the PPI assay can be difficult to implement.

More recent studies assessing habituation in zebrafish larvae have begun to explore alternative behavioural read-outs of startle responses, including locomotion which can be done at lower camera frame rates, or general motion detection in adult fish for sensory gating experiments (Beppi et al., 2021; Kirshenbaum et al., 2019) . However, such simpler approaches have not yet been created and validated for a PPI-type paradigm in larvae or in context with schizophrenia-relevant pharmacological modulation. In this report, we present the establishment of a locomotion-based PPI (LB PPI) assay based only on measuring locomotion as a read-out for the startle response in zebrafish larvae. We furthermore show that it is possible to observe a schizophrenia-reminiscent PPI reduction by administration of the glutamatergic NMDA receptor antagonist, MK-801.

The LB PPI assay was set-up using the Zantiks MWP ( https://zantiks.com/ ), which is an automated system containing a low-speed camera at 30 frames per second and 720 x 540 pixels image size. To produce the startle stimuli, vibrations were delivered using an in-built motor which is script-controlled. To generate a reliable LB PPI protocol using this system, we optimised two parameters, namely, vibration intensity and inter-stimulus interval (ISI, the time between startle and prepulse vibrations).

Based on previous literature, vibrations eliciting a variation in SLC startle responses in zebrafish larvae are between 100-1000 Hz (Beppi et al., 2021; Bergeron et al., 2015; Burgess & Granato, 2007) . As such, we set the frequency of both the startle and prepulse stimuli to 200 Hz. To deliver the vibration, the motor was set to move by either 1.8°(1 full step), 0.9° (1/2 step), 0.45°(1/4 step), or 0.225°(1/8step) in 4 x 5 ms clockwise and anti-clockwise movements. To evaluate if larvae would respond differently to different motor step sizes, eight single pulses (two at each step size), spaced at 5-minute inter-trial intervals (ITIs) were presented to larvae. Previous papers have used a minimum of 15 seconds (Burgess & Granato, 2007) and a maximum of 15 minutes ITI (Beppi et al., 2021) , thus we felt confident that no habituation to startle stimuli would be observed at 5 minute ITI. Startle response magnitude and probability were measured using distance moved (locomotion) per 100 msec time bin.

Larvae showed a graded startle magnitude at varying pulse intensities, as is shown in the raw locomotion trace from -500 ms to +500 ms from the startle pulse onset ( Figure 1A ). The response magnitudes for responder larvae only (those fish that show a startle response within 100 ms of the vibration) was analysed across pulse intensities, using a one-way ANOVA (F(3, 279) = 10.67, p < 0.0001; Figure 1B ). Larvae exhibited a significantly greater startle magnitude to 1 step compared to ½ step (p < 0.0001), ¼ step (p = 0.0196) and 1/8 step (p = 0.0003). This is likely reflective of the graded LLC response at different vibration intensities (although the lower frame rate of our camera set-up prevents absolute confirmation of this). However, PPI in the larval zebrafish has previously been defined as a difference in SLC probability when the startle stimulus is preceded by a prepulse stimulus (Burgess & Granato, 2007) . As such, we explored whether we could detect changes in response *probability* across the different pulse intensities for all larvae ( Figure 1C ). A one-way ANOVA showed that response probability varied across pulse intensities (F(3,380) = 22.03, p < 0.0001). Notably, larvae exhibited a significantly greater response probability at 1 full step compared to ½ step (p < 0.0001) and 1/8 step (p<0.0001), and significantly lower response probability at 1/8 step compared to 1/2 step (p = 0.0230) and ¼ step (p<0.0001). On account of these findings, it is possible to observe changes in the probability of the larval startle response at varying pulse intensities, likely reflective of increased probability of SLC startle at higher pulse intensities. In summary, we were able to observe a change in startle response read-out based on startle magnitude, and this mirrored response probability, based on only locomotion recordings.

When translating these findings into a full two-pulse PPI paradigm, we wanted to explore whether it was possible to observe a modulation of the locomotion response by introducing a preceding lower intensity prepulse before the startle stimulus. Based on the above findings, the startle vibration was set at 200 Hz with one full motor step, and the prepulse vibration set to 200 Hz with 1/8 motor step. These startle and prepulse vibrations were applied to all subsequent PPI assays. Although both response magnitude and response probability were modulated by these stimulus intensities, it was unclear how this would manifest by applying PPI measurements and calculations. This is because both SLC and LLC startle responses are captured in 100 ms time bins and it has not so far been determined how these startle responses might equate with locomotion measurements. As such, we continued to consider both response magnitude and response probability, alongside the locomotion-based PPI measurement.

When running the LB PPI assay in full, we varied the inter-stimulus interval (ISI) from 100 to 1000 ms in a between-subjects experimental design ( Figure 1D ). This ISI range is consistent with earlier reports showing that the ISI greatly impacts PPI between 30 to 3000 ms, with the highest PPI percentages being observed at intermediate ISIs of around 300-500 ms (Bergeron et al., 2015; Burgess & Granato, 2007) . In total, eight vibration trials were delivered in each PPI assay. Startle pulse alone and prepulse/startle trials were evenly interspersed with a total of four of each trial type ( Figure 1D ).

When observing the raw locomotion with a 300 ms ISI, there is a sharp increase in distance travelled within 100 ms upon presentation of the startle stimulus on startle only trials ( Figure 1E ). However, when the prepulse preceded the startle stimulus (on prepulse/startle trials), there was a much-attenuated increase in distance travelled in response to the startle stimulus. Using only distance travelled, a clear PPI effect was observed in responder larvae at all ISI values ( Figure 1F ; see Methods for calculation of LB PPI). A one-way between-subjects ANOVA of percentage PPI for each ISI was significant (F(5, 173) = 3.53, p = 0.0046), and post-hoc t-tests revealed that this was driven by a significantly lower percentage PPI between 200 ms ISI and 300 ms ISI (p = 0.0254), 400 ms ISI (p = 0.0469) and 500 ms ISI (p = 0.0127, Figure 1F ).

In addition to just calculating locomotion-based PPI, we also considered how response magnitude and response probability were affected by the prepulse/startle trials. Considering response magnitude of responder larvae on each startle trial type and ISI ( Figure 1G ), a two-way ANOVA was significant for trial type (F(1, 254) = 19.78, p<0.0001), where an increase in response magnitudes on the startle trial compared to the prepulse/startle trial was observed across all ISIs. There was no main effect of ISI (F(5,254) = 1.19, p = 0.3142) and no interaction between ISI and trial type (F(5,254) = 2.11, p = 0.0646). When analysing response probability of all larvae for the trial types and ISIs ( Figure 1H ), there was a main effect of trial type (F(1,506) = 104.10, p < 0.0001), which was again due to a higher response probability on startle trials compared to prepulse/startle trials (two-way ANOVA). In addition, there was also an effect of ISI (F(5, 506) = 5.29, p < 0.0001), where response proportion was significantly greater with an ISI of 300 ms compared to 100 ms, 400 ms, 500 ms and 1000 ms (p < 0.05 for all) and significantly greater again at 200 ms versus 400 ms and 500 ms (p<0.01). Finally, there was an interaction between ISI and trial type (F(5,506) = 4.84, p = 0.0002). Here, key post-hoc analyses showed response probabilities were significantly greater in startle alone trials compared to prepulse/startle trials (all p < 0.05; Figure 1H ). As such, both response magnitude (mostly driven by LLC startles) and response probability (mostly driven by SLC startles) contribute to a locomotion-based PPI. Though it seems that decreasing response probability on prepulse/startle trials compared to startle alone trials is the more consistent driver behind LB PPI, which is in line with what has been observed in standard measures of SLC for PPI. These findings led us to choose 300 ms as the ISI of the final LB PPI assay as the goal was to use an ISI with a strong and consistent reduction in response magnitude and response probability.

In order to show the LB PPI is applicable to neuropsychiatric models in the zebrafish, we tested whether PPI percentage could be modulated by dizocilpine (MK-801), a non-competitive NMDA receptor antagonist, commonly used to pharmacologically create animal models of schizophrenia. Larvae were exposed to the drug for the full duration of the assay while controls were exposed to a DMSO-matched solution. Consistent with previous studies, the LB PPI percentage of responder larvae was greatly reduced in MK-801-exposed larvae (29.48% ±12.04% SEM) compared to controls (77.40% ±7.25% SEM) (t(126) = 3.21, p = 0.0017; Figure 1I ). In addition, a two-way ANOVA of responder larvae response magnitudes for trial type and drug condition showed no main effect of trial type (F(1, 633) = 0.0161, p = 0.8993), but a strong effect of drug condition (F(1, 126) = 31.47, p < 0.0001), caused by significant elevation in startle response magnitude for the MK-801 group on both types of trials ( Figure 1J ). However, there was no interaction between startle condition and drug condition (F(1,63)=1.78, p = 0.1860). With regards to response proportion ( Figure 1K ), there was a main effect of trial type (F(1,157) = 54.30, p<0.0001), where response probability was significantly greater on startle alone trials than prepulse/startle trials, and a main effect of drug condition (F(1,157) = 33.36, p < 0.0001), where startle probability was significantly greater in MK-801-treated larvae compared to DMSO controls ( Figure 1K ). Thus, both response magnitude and probability were increased uniformly across trial types by MK-801 administration, and across both drug conditions response probability is significantly greater on startle alone trials compared to prepulse/startle trials. It is thus likely a combination of both altered response probability and magnitude is driving the decrease in LB PPI for MK-801-treated larvae.

In summary, the data here show that a locomotion-based PPI paradigm can be conducted using larval zebrafish, using low frame rate cameras. Consistent to what has been observed when measuring c-bend startles in larval zebrafish, our LB PPI assay shows a reduction in larval startle probability in prepulse/startle trials, based on measuring locomotion only (Bergeron et al., 2015; Burgess & Granato, 2007) . In addition, the startle magnitude is also decreased on prepulse/startle trials compared to startle alone trials, which can be used as a measure for PPI.

Our results further showed reductions in PPI after administration of the NMDA receptor antagonist MK-801, in line with previous studies using different PPI startle readouts (Bergeron et al., 2015; Wolman et al., 2011) . This is particularly interesting as strong links have been made between glutamatergic abnormality, particularly in the hippocampal CA1/CA3 regions in mammals, and schizophrenia risk/symptoms (Briend et al., 2020; Demjaha et al., 2014; Egerton et al., 2018; Park et al., 2021; Uno & Coyle, 2019) . There is also a direct translatability in the PPI response between animal models and humans. Notably, the ISI parameters of the PPI assay for larval zebrafish assays are nearly identical to those used with human participants, allowing direct comparability between endophenotypes (San-Martin et al., 2020; Swerdlow et al., 2006) .

High temporal resolution PPI that measures larval c-bends are useful for more fine-grained analysis of neuronal circuits in motor movement and therefore might be still selected for certain experimental paradigms (Eaton et al., 1977; Liu & Fetcho, 1999; Medan & Preuss, 2014) . However, our LB PPI assay is clearly suitable to provide a tool for rapid assessment of sensory gating in larvae. This is particularly useful for conducting high-throughput screens of disorder-associated genes and novel compounds, one of the strengths of the zebrafish larval system.

## Methods

*Zebrafish Husbandry and Maintenance*

Zebrafish ( *Danio rerio* ) were maintained in accordance with the Animals (Scientific Procedures) Act 1986 under license from the United Kingdom Home Office (PP7266180). AB strain wildtype zebrafish larvae were used at 6 days post-fertilisation (dpf). Embryos and larvae were maintained at 28.5°C on a 14 h ON/10 h OFF light cycle in methylene blue (methylthioninium chloride)/1X Danieau’s medium (NaCl 174 mM, KCl 2.1 mM, MgSO4 1.2 mM, Ca(NO _3_ ) _2_ ) 1.8mM, HEPES 15mM, pH7.6).

*Drug Preparation*

MK-801 hydrogen maleate (dizocilpine) was dissolved in 100% DMSO and then further diluted to 100 µM of MK-801 and 0.01% DMSO. The control comparison solution was 0.01% DMSO in Danieau’s medium.

*Prepulse Inhibition (PPI) Assay*

All experiments were carried out between 12:00-17:00 in a Zantiks MWP behavioural box ( https://zantiks.com/ ). Larvae were habituated to the procedure room 30 minutes prior to the experiment. All larvae were then placed into a 96-well plate for PPI vibrations and locomotion tracking. Each well was filled with 200 µl of 1X Danieau’s medium. In the MK-801 experiment, larvae were placed in 200 µl of DMSO-matched control or 100 µM MK-801.

Startle stimuli were delivered using a motor mechanism that could produce script-controlled vibrations. The motor was programmed to move in alternating directions, clockwise and anti-clockwise for a total of 4 step movements, each lasting 5 ms at a 200 Hz frequency. This was consistent in all single-pulse and PPI assays shown here. In the process of optimising the PPI assay, the vibration stimuli were presented at varying step sizes (1, ½, ¼ and 1/8 step) where 1 full step size was a 1.8° motor movement. The inter-stimulus interval (ISI) was varied between 100 ms and 1000 ms. In the final PPI assay (see Figure 1D ), 8 stimulus trials occurred with a 5-minute inter-trial interval (ITI) and a 300 ms ISI. Trial types alternated randomly between startle stimulus alone and prepulse/startle stimulus trials to avoid habituation to trial types. The startle stimulus was 1 step size and the prepulse stimulus was 1/8 step.

*Data Analysis*

Data was extracted using Matlab (R2022b), and statistically analysed and graphed using GraphPad Prism (version 10). Prior to data analysis, larvae showing 0 mm locomotion +/- 500 ms around the startle vibration were excluded from further analyses, as was data that was subject to technical errors. PPI percentage was calculated using the following equation:

%PPI = ( ( Startle_alone − ( Prepulse Startle ) ) / Startle_alone ) * 100

“Startle alone” was the mean locomotion at +100 ms in startle alone trials and “Prepulse/Startle” is the mean locomotion at +100 ms in prepulse/startle trials ( Figure 1E ). 0 ms was the time bin that the startle pulse was delivered in (see Figure 1E ). For analyses of % PPI and startle magnitude, larvae were classified as a “responder larvae” if they showed locomotion of >0 mm in the +100 ms time bin (i.e., a startle response). For analyses of startle probability, all larvae were included. N-numbers for larvae included in each experiment and analysis type can be found below in Table 1. In the case where post-hoc analyses were conducted, Šidák’s or Tukey’s multiple comparison corrections were applied.

***Table 1.*** *N* -numbers per group in each vibration/PPI experiment.

**Table:** 

	***N* -total per group (after exclusions) **	***N* - responder larvae per group (startle) **	***N* - responder larvae per group (pp/startle) **
** Single Pulses ( Figure 1A -C) **	96 (within-subject)	1 step: 78 ½ step: 78 ¼ step: 77 1/8 step: 50	N/A
** PPI assay ( Figure 1E -H) **	100 ms: 72 200 ms: 37 300 ms: 40 400 ms: 34 500 ms: 33 1000 ms: 38	100 ms: 47 200 ms: 28 300 ms: 34 400 ms: 18 500 ms: 24 1000 ms: 28	100 ms: 30 200 ms: 20 300 ms: 7 400 ms: 6 500 ms: 6 1000 ms: 9
** MK-801 assay ( Figure 1I -K) **	DMSO-control: 74 MK-801: 85	DMSO-control: 57 MK-801: 71	DMSO-control: 18 MK-801: 48
